# Topographic de-adhesion in the viscoelastic limit

**DOI:** 10.1098/rsif.2022.0598

**Published:** 2023-01-11

**Authors:** Nhung Nguyen, Eugenio Hamm Hahn, Sachin Velankar, Enrique Cerda, Luka Pocivavsek

**Affiliations:** ^1^ Department of Surgery, The University of Chicago, Chicago, IL, USA; ^2^ Departamento de Física, Facultad de Ciencia, Universidad de Santiago de Chile (USACH), Santiago, Chile; ^3^ Department of Chemical Engineering, University of Pittsburgh, Pittsburgh, PA, USA

**Keywords:** topography, wrinkle, viscoelastic, biofoulants, de-adhesion, finite-element simulations

## Abstract

The superiority of many natural surfaces at resisting soft, sticky biofoulants have inspired the integration of dynamic topography with mechanical instability to promote self-cleaning artificial surfaces. The physics behind this novel mechanism is currently limited to elastic biofoulants where surface energy, bending stiffness and topographical wavelength are key factors. However, the viscoelastic nature of many biofoulants causes a complex interplay between these factors with time-dependent characteristics such as material softening and loading rate. Here, we enrich the current elastic theory of topographic de-adhesion using analytical and finite-element models to elucidate the nonlinear, time-dependent interaction of three physical, dimensionless parameters: biofoulant’s stiffness reduction, the product of relaxation time and loading rate, and the critical strain for short-term elastic de-adhesion. Theoretical predictions, in good agreement with numerical simulations, provide insight into tuning these control parameters to optimize surface renewal via topographic de-adhesion in the viscoelastic regime.

## 1. Introduction

**Figure 1.**
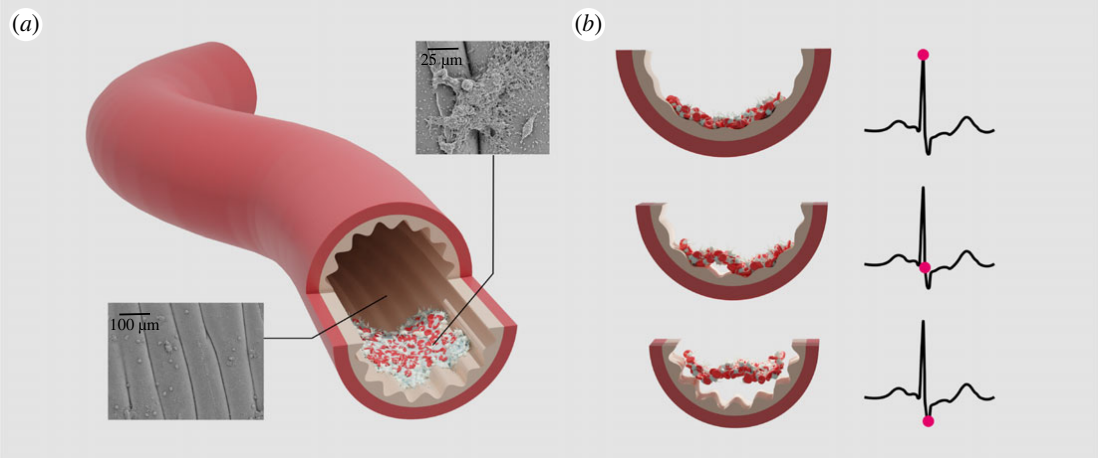
Topography-driven delamination motivated by dynamically actuated wrinkled surface in artery. (*a*) A three-dimensional view of biofoulants attached to the arterial luminal wrinkles. The SEM images show that the arterial wrinkle surface is covered with platelets and thromboses at an early stage. The schematic drawing of the artery provides a continuum model for their interaction that has been proposed in previous works [8–10] at the stage where biofoulants grow and expand over multiple wrinkle wavelengths. (*b*) Cross-sectional views of the postulated detachment process of biofoulants from the wrinkled topography at different levels of actuated pulse pressure through the cardiac cycle illustrated in black on the right. During systole (top), the high pressure distends the artery and flattens the luminal wrinkling. As the cardiac cycle progresses, the arterial pressure drops gradually until the diastolic pressure (bottom) is reached. As the pressure decreases, the artery contracts and the amplitude of the wrinkles grows. The cardiac cycle then repeats when the left ventricle again contracts pumping blood into the arterial system. Current attempts with experiment and computational modelling focus on elastic behaviours of the biofoulant and show a scaling dependence of the critical surface curvature on the surface energy, bending stiffness and topographic geometrical wavelength [8].

Many natural surfaces including airways, arteries and intestines have dynamically actuated geometries [1–11]. Specifically, pulse pressure in arteries is believed to drive the luminal arterial geometry between low and high curvature states, generating an actuating topography [8–11]. Figure 1 schematically demonstrates the change in arterial topography during the cardiac cycle, with a flat luminal surface at systole (figure 1*b*, top) and a wrinkled surface at diastole (figure 1*b*, bottom). The existence of these states has been experimentally validated *ex vivo* in fresh arterial segments in a previous work [12]. Such topographic variations may play an important role in keeping these surfaces clean from biofouling on macroscopic scales and potentiate nature’s multi-scale anti-fouling strategies at complex interfaces. Inspired by these natural phenomena, a new de-adhesion mechanism, using dynamic surface topography, was discovered: topography-driven delamination (figure 1), where foulant deformation, driven by evolving surface curvature, generates an energy release mechanism by balancing elastic energy with adhesion strength [6–10]. Thus far, topography-driven delamination has only been explored in the idealized elastic foulants, where all stored elastic energy is available to drive surface renewal once a critical surface curvature, *κ*_*c*_, is reached [8].

Biofoulants, such as platelets, thrombus, biofilms and bacteria, are complex, dynamic materials. In order to integrate topographic anti-fouling strategies into biomaterials and medical devices with specific engineering design limits with given loads and achievable actuation strains, topography-driven delamination must be understood in the viscoelastic regime that more accurately represents the material properties of biological foulants [2,8,13–17]. Viscoelasticity allows a continuous softening of material properties, a dependence on loading history and an intrinsic mechanism for dissipating elastic energy within a stressed material [18,19]. Since topography-driven delamination is an energy release mechanism where available foulant elastic energy drives interfacial fracture, the existence of a competing dissipation mechanism, intrinsic to the material, enriches the problem substantially, compared with the elastic limit.

**Figure 2.**
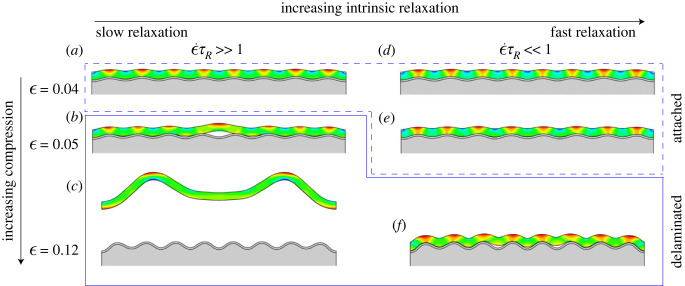
Delamination processes under increasing applied strain ϵ of a slowly relaxing viscoelastic foulant layer (*a*–*c*) when compared with a fast relaxing viscoelastic foulant layer (*d*–*f*). The latter conforms more stably to the wrinkled topography and requires a higher strain ϵ=0.12 (*f*) than the former ϵ=0.05 (*b*) to cause its detachment.

A model system with a thin foulant on a bilayer composed of a thin elastic film on a soft, thick elastic substrate is adopted from the previous work [8]. The system is subjected to continuous loading on the two ends with the deformation profile $\epsilon =\dot{\epsilon }t$, where *t* is the loading time. If the foulant behaves elastically, details of the delamination process have been presented in this previous work. In our Results section, we will provide detail calculations for the viscoelastic foulant. Figure 2 shows a striking difference between the detachment process of an elastic (or equivalently, a viscoelastic foulant layer with a large intrinsic relaxation time *τ*_*R*_ relative to the intrinsic loading rate $\dot{\epsilon }$) and a viscoelastic foulant layer with a short relaxation time scale (see also electronic supplementary material, video S1). In both cases, the foulant layers initially follow the wrinkled surface, which is the source of changing curvature *κ* = *A*/*λ*^2^, where *A* is wrinkle amplitude and *λ* is wrinkle wavelength. They ultimately delaminate; however, the one with fast intrinsic relaxation, $\dot{\epsilon }\tau_{R}\ll 1$, remains attached until higher critical curvatures (critical strains). These examples show that the presence of significant viscoelasticity, on the time scale of loading, stabilizes the foulant layer/substrate interface. Of note, the nominal compressive critical strain more than doubles from approximately  5% to  12% in these two cases.

For optimal design of self-cleaning surfaces in contact with viscoelastic biofoulants, the interaction of the time-dependent characteristics of the viscoelastic foulant layer with the geometry set by the wrinkled topography and the surface energy needs to be understood, especially for dynamic topography-driven anti-fouling strategies in biomaterials and medical devices [6,7,9,10]. In this paper, we tackle the complex interaction between foulant layer’s viscoelasticity, rate of topographic changes, mechanical instability and surface adhesion in the wrinkle-induced delamination model [8]. Using analytical methods based on energy minimization [8,18,20–28] and finite-element analysis with cohesive zone modelling [8,25,29–37], we reduce the parameter space that controls the delamination of a thin, viscoelastic foulant layer from a wrinkled surface to three physical dimensionless parameters: magnitude of foulant layer’s relaxation, rate of material relaxation relative to loading rate and the critical strain for delamination in the short-term (instantaneous elastic) limit. The last parameter incorporates the surface energy, the bending stiffness of the foulant layer and the surface geometry via the wrinkle wavelength. Our analytical model for viscoelastic topographic de-adhesion is able to collapse numerical data from a large range of the dimensionless parameter space. Our analysis provides insight into viscoelastic delamination for geometrically nonlinear interfaces that inevitably exist in biological systems and for artificial materials incorporating topography as self-cleaning strategies [6–10].

## 2. Results

### 2.1. Scaling analysis

**Figure 3.**
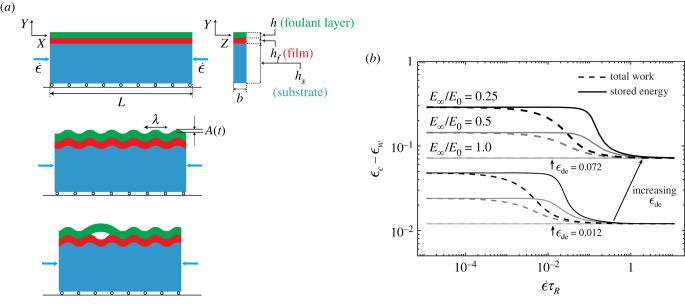
Compression of a viscoelastic foulant layer attached to a bilayer for studying wrinkle-induced delamination. (*a*) Schematics of a thin viscoelastic foulant layer attached to a bilayer system composed of a thin film on top of a substrate subjected to increasing compression. The bilayer wrinkles, which induces a wrinkle pattern on the foulant layer. As the wrinkle curvature increases *κ* = *A*/*λ*^2^, the foulant layer starts to de-adhere from the bilayer. Here, *A* and *λ* are the wrinkle amplitude and wavelength. (*b*) Energy balance approach shows the dependence of the critical strain on three controlling dimensionless parameters. Two representative values of $\epsilon_{\mathrm{de}}$ and three representative values of *E*_∞_/*E*_0_ are selected for plotting here. Note that when *E*_∞_/*E*_0_ = 1.0, the result of the elastic foulant layer [8] is reproduced as shown by the horizontal, flat curves. In these cases, the critical strain is equal to $\epsilon_{\mathrm{de}}$ and is independent of $\dot{\epsilon }\tau_{R}$.

A viscoelastic foulant layer (adherent biofoulant) of thickness *h* is attached to the surface of an elastic bilayer designed to mimic the multi-layered structure of the arterial wall [8,12]. The resultant tri-layer system is subjected to compression as shown in figure 3*a*. Under increasing applied compression on the two ends and assuming plane strain in the orthogonal *z*-direction, the bilayer composed of a stiff film of thickness *h*_*f*_ attached to a soft substrate of thickness *h*_*s*_ ≫ *h*_*f*_, undergoes wrinkling with increasing wrinkle amplitudes as described previously [3,8,38–43]. Specifically, the critical strain for wrinkle onset is set by the mismatch stiffness between the film and the substrate: $\epsilon_{w}∼{\left(E_{s}/E_{f}\right)}^{2/3}$ where *E*_*s*_ and *E*_*f*_ are the substrate and the film moduli, respectively, and *E*_*f*_ ≫ *E*_*s*_. Surface topography is set by its curvature *κ* = *A*/*λ*^2^, which depends on wrinkle wavelength *λ* ∼ *h*_*f*_ (*E*_*f*_/*E*_*s*_)^1/3^ and amplitude $A∼\lambda \sqrt{\epsilon -\epsilon_{w}}$, where ϵ is the applied nominal strain. This wrinkle pattern is not affected by the foulant layer because the foulant layer is significantly softer than the constituents of the bilayer [8]. To reduce the effect of the pre-wrinkled state on the subsequent delamination process, we study the regime where the wrinkle onset strain $\epsilon_{w}$ is small, thereby $A∼\lambda \sqrt{\epsilon }$. As a critical curvature $\kappa_{c}∼A_{c}/\lambda^{2}∼\sqrt{\epsilon_{c}}/\lambda$ is reached, the viscoelastic foulant layer starts to de-adhere from the bilayer surface. In the elastic limit for the foulant layer solved by Pocivavsek *et al.* [8], no time-dependent quantity enters the solution $\epsilon_{c}=\epsilon_{\mathrm{de}}=a\lambda^{2}G/B$, where *G* is the adhesion energy, *B* = *Eh*^3^/12(1 − *ν*^2^) is the bending stiffness of the elastic foulant layer (*ν* is the Poisson’s ratio of the foulant layer) and *a* is a numerical pre-factor. In the current case of viscoelastic foulant layer, the intrinsic relaxation time of the material interacts with the time scale set by the rate of topographic loading to give rise to a time-dependent delamination condition [44]. Here, for the simplest viscoelastic foulant layer, the relaxation is described using a single term Prony series: $E(t)=E_{\infty }+(E_{0}-E_{\infty })\;e^{-t/\tau_{R}}$, where *τ*_*R*_ is the relaxation time and *E*_∞_ ≤ *E*_0_ are the long-term and instantaneous elastic stiffnesses of the foulant layer, respectively. A mechanical analogue for this representation is a Maxwell model with two springs of stiffnesses *E*_∞_ and *E*_0_ − *E*_∞_ and a dashpot to dissipate energy (see electronic supplementary material, appendix 1). With this decaying function of the modulus, a simple mathematical extension of the result of Pocivavsek *et al.* [8] for compression applied at a constant rate $\epsilon =\dot{\epsilon }t$ is to substitute *E*(*t*) into the scaling law derived in the elastic limit, which leads to the identity

2.1 $$\epsilon_{c}=\frac{a\lambda^{2}G/B_{0}}{1-\beta (1-e^{-\epsilon_{c}/(\dot{\epsilon }\tau_{R})})}.$$

In terms of time scales, this equation is written as

2.2 $$\frac{t_{c}}{\tau_{R}}=\frac{\tau_{\mathrm{de}}/\tau_{R}}{\left[1-\beta (1-e^{-t_{c}/\tau_{R}})\right]},$$

where $\epsilon_{c}=\dot{\epsilon }t_{c}$ and *t*_*c*_ are the critical strain and delamination time, respectively; *β* = (*B*_0_ − *B*_∞_)/*B*_0_ = (*E*_0_ − *E*_∞_)/*E*_0_ is the fraction of the stiffness reduction in the foulant layer, with *B*_0_ = *E*_0_ *h*^3^/12(1 − *ν*^2^) and *B*_∞_ = *E*_∞_*h*^3^/12(1 − *ν*^2^) providing bending stiffnesses of the foulant layer at the instantaneous and long-term elastic limits, respectively; and $\tau_{\mathrm{de}}=\epsilon_{\mathrm{de}}/\dot{\epsilon }$ is the characteristic time for delamination in the instantaneous elastic limit.

Three physical dimensionless parameters emerge from these equations: *E*_∞_/*E*_0_ (magnitude of the foulant layer’s relaxation), $\dot{\epsilon }\tau_{R}$ (rate of material relaxation relative to loading rate, which is similar to the Weissenberg number used by rheologists to quantify viscoelastic effects [45]) and $\epsilon_{\mathrm{de}}$ (critical delamination strain at the instantaneous elastic limit, which depends on wrinkle wavelength, adhesion strength and the instantaneous elastic modulus of the foulant layer as quantitatively described by the elastic model of Pocivavsek *et al.* [8]). The ratio between the last two parameters *τ*_de_/*τ*_*R*_ is a control parameter for the system: when *τ*_de_/*τ*_*R*_ ≪ 1 we expect from the balance in equation (2.2) that *t*_*c*_/*τ*_*R*_ ≪ 1. It implies that the denominator is approximately equal to one such that *t*_*c*_ ≈ *τ*_de_. Similarly, when *τ*_de_/*τ*_*R*_ ≫ 1 the same balance gives *t*_*c*_/*τ*_*R*_ ≫ 1 and the result $t_{c}\approx \tau_{\mathrm{de}}/(1-\beta )=a\lambda^{2}G/(\dot{\epsilon }B_{\infty })$. Thus, delamination is controlled by the bending stiffness *B*_0_ for *τ*_de_/*τ*_*R*_ ≪ 1 and the opposite limit corresponds to delamination controlled by *B*_∞_.

### 2.2. Energy analysis

Though the above mathematical approach indicates the presence of important dimensionless parameters controlling this complex viscoelastic system and their nonlinear coupling, to obtain a physical mechanism driving delamination in this system, we employ an analysis based on energy minimization. In the case of a purely elastic foulant layer without delamination, the work performed by the wrinkled surface generates stored elastic energy in the foulant layer $\dot{W}=\dot{U}$, where the total work injected into the system is $\dot{W}=\int_{V}\sigma \dot{\epsilon }\;dV$ and σ=Eϵ. In the case of a viscoelastic foulant layer, part of the total work is stored and the other part is dissipated through viscous relaxation: $\dot{W}=\dot{U}+\dot{D}$. Here again $\dot{W}=\int_{V}\sigma \dot{\epsilon }\;dV$ but the stress and strain are divided in two parts: $\sigma =E_{\infty }(\epsilon^{e}+\epsilon^{v})+(E_{0}-E_{\infty })\epsilon^{e}$ and $\epsilon =\epsilon^{e}+\epsilon^{v}$, with $\epsilon^{e}$ being the elastic strain and $\epsilon^{v}$ being the viscous strain. Using a mechanical analogue of linear viscoelasticity (see electronic supplementary material, appendix 1), the stored elastic energy of the foulant layer is derived as

2.3 $$U=\frac{E_{\infty }}{2}\int_{V}\;dV{\left(\epsilon^{e}+\epsilon^{v}\right)}^{2}+\frac{E_{0}-E_{\infty }}{2}\int_{V}\;dV{\left(\epsilon^{e}\right)}^{2}.$$

Note that for *t* ≪ *τ*_*R*_, no viscous dissipation has yet occurred as no viscous strain has had time to develop, thus *D* = 0, $\epsilon^{v}=0$ and $U=(E_{0}/2)\int_{V}\;dV{\left(\epsilon^{e}\right)}^{2}$. On the other hand, for *t* ≫ *τ*_*R*_, the material has exhausted all possible sources of viscous dissipation, thus the foulant layer’s elastic strain $\epsilon^{e}=0$ and $U=(E_{\infty }/2)\int_{V}\;dV{\left(\epsilon^{v}\right)}^{2}$. Since the total strain $\epsilon =\epsilon^{e}+\epsilon^{v}$, we see in the two limits the available energy is simply that of the purely elastic case with the two moduli *E* = *E*_0_ and *E* = *E*_∞_ in the single term Prony series. In other words, a physical interpretation of the stored energy is the elastic energy stored in the two elastic springs of the Maxwell model [18]. At the instantaneous limit *t* ≪ *τ*_*R*_, *U* and *W* reduce to the bending energy for the elastic case with modulus *E*_0_. At the long-term limit *t* ≫ *τ*_*R*_, *U* and *W* have the same form of the elastic bending energy but with modulus *E*_∞_. However, in the intermediate regime between these two limits, the difference between *U* and *W* arises due to active viscous dissipation in this system (see detailed derivations in electronic supplementary material, appendix 2). It is in this active regime, where two time-dependent mechanisms interact, that the analysis becomes interesting and complex.

To drive fracture, a balance between the energy available in the system and the surface energy is required. For a conservative system, d*W* = d*U* and a balance between the stored energy and the surface energy d(*U* + *U*_*S*_) = d(*W* + *U*_*S*_) = 0, where *U*_*S*_ = *Gl* and *l* is the crack length, is widely used to compute variations of the crack length to study fracture under displacement controlled conditions [8,23–26,46]. However, for a dissipative system, due to the presence of the material’s dissipation energy, two arguments are presented in the literature for the source of energy to predict fracture. One approach balances the total work *W* against the surface energy as a condition for crack propagation [18,27]: d(*W* + *U*_*S*_) = 0. A second approach assumes that only the stored energy *U* is the amount of energy available for fracture [28]: d(*U* + *U*_*S*_) = 0. Note that both approaches are equivalent for a conservative system. The correct energy is debatable, and more accurate experimental data and understanding of other possible sources of energies that might play a role in real systems such as dynamic effects are needed to resolve this conflict. Yet, these two approaches provide insights into two limiting cases for the energy available for driving fracture in a non-conservative, viscoelastic system. In particular, the first one considers the largest possible amount of energy while the second one considers the smallest possible amount of energy available to overcome surface energy and cause fracture to occur. Thus, in order to physically understand the emergence and coupling of the three dimensionless parameters and how they govern the delamination of the viscoelastic foulant layer from the wrinkled surface, we use both approaches in our analytical models and compare them with the results obtained from numerical simulations.

Specifically, from the imposed wrinkled topography, the height of the mid-plane of the foulant layer is *h*_*m*_(*x*,*t*) = *A*(*t*)sin(*kx*), where $t=\epsilon /\dot{\epsilon }$ and *k* = 2*π*/*λ* is the wavenumber. The curvature is computed as $\kappa =\partial_{x}^{2}h_{m}(x,t)=-k^{2}A(t)\sin(kx)$ giving the bending strain *γ* = *κy* at a point located at 0 ≤ *x* ≤ *L* along the length *L* and at a distance of *y* to the neutral axis (the mid-plane) of the thin foulant layer. Thus, the strain in the foulant layer is computed as *γ* = −*yk*^2^*A*(*t*)sin(*kx*) (see electronic supplementary material, appendix 2). Neglecting the small compressive strain prior to buckling, the viscous strain in the foulant layer becomes $\gamma^{v}=(1/\tau_{R})\int_{0}^{t}\gamma (\tau )\;e^{-(t-\tau )/\tau_{R}}\;d\tau$ and the elastic strain is *γ*^*e*^ = *γ* − *γ*^*v*^. Both approaches lead to nonlinear, time-dependent equations whose solutions give the critical time (equivalently critical strain) to trigger delamination

2.4 $$\frac{t_{c}}{\tau_{R}}=\frac{\tau_{\mathrm{de}}/\tau_{R}}{\left[1-\beta P_{\pm }(t_{c}/\tau_{R})\right]},$$

where $P_{-}(z)=(1/z)\int_{0}^{z}dw(F(w)/\sqrt{w})$ and $P_{+}(z)=1-$ $(1-{\left(F(z)/\sqrt{z})\right)}^{2}$ are obtained using the total work *W* and stored energy *U*, respectively, with $F(z)=\int_{0}^{w}\sqrt{y}\;e^{-(w-y)}\;dy$. Both functions *P*_±_ are such that 0 < *P*_±_ < 1 and *P*_+_(*z*) > *P*_−_(*z*) so that 1/[1 − *βP*_+_(*z*)] > 1/[1 − *βP*_−_(*z*)] for 0 < *β* < 1 (see electronic supplementary material, appendix 2). Thus, the critical strain for delamination is always larger for the stored energy than the total work. This agrees with physical intuition, since in the case of the stored energy, only the elastic energy at any given time is available to drive fracture. However, in the case of total work, some of the dissipated energy may have gone into fracture. As compared with the empirical derivation in equation (2.2), the solutions for critical time *t*_*c*_ (or equivalently strain $\epsilon_{c}$) obtained from the energy balance approach involve more complex nonlinear time-convolution functions *P*_±_. Nevertheless, they again reveal the dependence of *t*_*c*_ (or $\epsilon_{c}$) on three physical dimensionless quantities. This further confirms the important role of these dimensionless parameters in controlling the wrinkle-induced delamination process of the viscoelastic foulant layer. Note also that in the elastic limit *E*_0_ = *E*_∞_, thus *β* = 0, the critical strain for delamination becomes the same $\epsilon_{\mathrm{de}}=\dot{\epsilon }\tau_{\mathrm{de}}=a\lambda^{2}G/B$ as presented in the previous work that focused on elastic foulant where the numerical scaling pre-factor *a* is used in the scaling law (see equation (30) in electronic supplementary appendix 2, figure S5 in electronic supplementary material, appendix 3 and [8]).

The wrinkling strain $\epsilon_{w}$ is assumed to be small, therefore the energy and relaxation in the foulant layer prior to wrinkling can be neglected in the above analysis. The effect of $\epsilon_{w}$, however, is taken as a shifting parameter to the solution obtained above as suggested in Pocivavsek *et al.* [8]: $\epsilon_{c}-\epsilon_{w}$. Shown in figure 3*b* are the critical strains for delamination obtained from solving equation (2.4) when the three physical dimensionless parameters are varied. While a decrease in *E*_∞_/*E*_0_ or in $\dot{\epsilon }\tau_{R}$ leads to an increase in the critical strain, a decrease in $\epsilon_{\mathrm{de}}$ reduces the critical strain. However, as the figure shows, the effects are highly nonlinear in the intermediate regime between the two elastic limits. In addition, smaller *E*_∞_/*E*_0_ values correspond to larger differences between the solutions using total work *W* and the stored energy *U* in the energy balance approach. When there is no intrinsic material relaxation, (*E*_∞_/*E*_0_ = 1), the solutions from both approaches coincide with the prior linearly elastic case [8]. Furthermore, in the two elastic limits $\dot{\epsilon }\tau_{R}\gg 1$ and $\dot{\epsilon }\tau_{R}\ll 1$, the two limiting elastic solutions $\epsilon_{c}-\epsilon_{w}=\epsilon_{\mathrm{de}}$ and $\epsilon_{c}-\epsilon_{w}=(E_{0}/E_{\infty })\epsilon_{\mathrm{de}}$ are obtained. Analysing the behaviour of the analytical functions *P*_±_(*z*) in equation (2.4) confirms the same limiting behaviour (see electronic supplementary material, appendix 2). It is important to note that $\epsilon_{c}-\epsilon_{w}$ does not simply increase linearly with the dimensionless parameter $\epsilon_{\mathrm{de}}$. Instead, we observe a right shift (in the arrow direction shown in figure 3*b*) for the transition from the instantaneous response to the other regimes which can be attributed to the nonlinear effect of *τ*_de_/*τ*_*R*_.

### 2.3. Finite-element analysis

**Figure 4.**
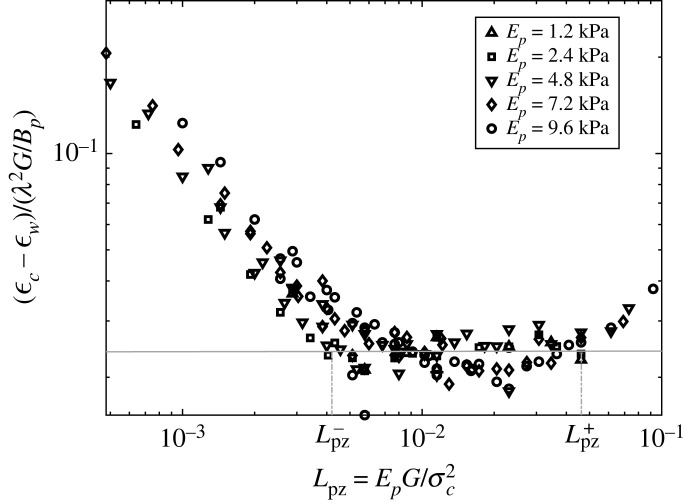
Normalized ratio (or the slope/pre-factor *a* in the scaling law: $\epsilon_{c}-\epsilon_{w}=a\lambda^{2}G/B_{p}$) versus *L*_pz_. Various values of *E*_*p*_ are examined. The flat line is a fit showing the linear scaling as predicted from scaling analysis which results in a slope of the same order as the one computed for the elastic foulant layer [8] (*a* ≈ 0.025, see electronic supplementary material, appendix 3).

To further study the roles of the dimensionless control parameters and their influence on $\epsilon_{c}$, as well as verify the trends observed from the analytical method, a detailed parametric study is performed using finite-element method (FEM) with cohesive zone model (CZM) implemented in Abaqus (Dassault Systèmes, MA) [47]. In this approach, traction separation laws are prescribed between the foulant layer and the film interface to study the delamination process [8,25,29–37]. Two input parameters, the cohesive strength *σ*_*c*_ and the fracture energy *G*, together with a damage law are necessary to describe the computational cohesive laws. In this study, bi-linear softening laws are employed to improve the numerical implementation in the previous study which employed linear elastic brittle laws [8] (see electronic supplementary material, appendix 3). The contributions of the two CZM parameters *σ*_*c*_ and *G* to the fracture process are also conveniently studied with this implementation. Solving equilibrium equations set by balancing the total energy, including contributions from both the foulant layer and the cohesive interface, allows the determination of $\epsilon_{c}$. In Abaqus, this solution process can be based on an implicit or explicit solver [47]. The implicit solver offers the advantage of solving this quasi-static problem without introducing additional dynamic energy. However, the presence and coupling of surface instability, material softening, contact conditions and interfacial delamination require tuning of various solver parameters and the introductions of certain artificial energies, such as damping, to resolve convergence issues of this iterative solution scheme. Therefore, in this study, we employ Abaqus dynamic explicit solver in order to conduct a parametric study with minimum adjustment of solver parameters over a large space of material properties and varying loading rates. Furthermore, in the CZM method, the process zone length along the interface $L_{\mathrm{pz}}=EG/\sigma_{c}^{2}$, which is the length over which CZM elements enter the degradation part of the traction separation law, affects the delamination mechanism in the problem. It has been shown in the literature for several classical interfacial geometries that *L*_pz_ has to be smaller than a characteristic length of the system for CZM to produce the same solution as the energy-based approach, such as *L*_pz_ < *h* for double cantilever beam, where *h* is the beam thickness [32,33], or *L*_pz_ < *L*^2^/(2*h*) for edge delamination, where *L*, *h* are the length and thickness of the layer [29]. The role of *L*_pz_ was not considered in prior topography-driven delamination work [8]. Thus, here we conducted a detailed sensitivity study for the effects of CZM parameters on $\epsilon_{c}$ in the instantaneous and long-term limits for the viscoelastic foulant layer. At these limits, the viscoelastic foulant layer can be treated as an elastic foulant layer with modulus *E*_*p*_ = *E*_0_ = *E*_∞_ and the scaling law becomes: $\epsilon_{c}=a\lambda^{2}G/B_{p}$, where *a* is a constant pre-factor [8]. Figure 4 plots the normalized ratio $\left(\epsilon_{c}-\epsilon_{w})/(\lambda^{2}G/B_{p}\right)$, which provides the parameter *a*, as a function of *L*_pz_ for different foulant layer’s modulus *E*_*p*_. For each foulant layer’s modulus, simulations with different sets of CZM parameters *σ*_*c*_ and *G* were performed.

**Figure 5.**
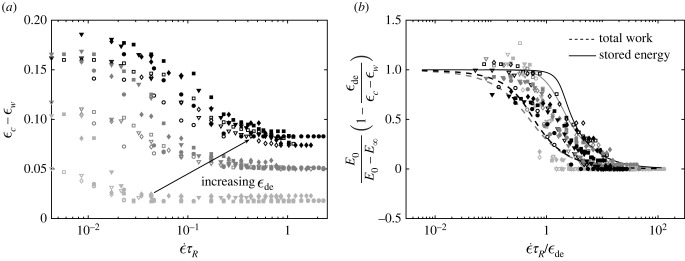
Finite-element results show how the three controlling dimensionless parameters affect the critical strain (*a*) and general fits to collapse numerical and analytical data (*b*). Four marker types (downward pointing triangle, square, diamond and circle) correspond to four loading rates $\dot{\epsilon }/{\dot{\epsilon }}_{\mathrm{el}}=0.75,1,1.5,2$, respectively, used in FE simulations, where ${\dot{\epsilon }}_{\mathrm{el}}$ is the loading rate adopted from the FE simulations in the previous study for elastic foulant layer [8] (see electronic supplementary material, appendix 3). Open and closed markers are for *E*_∞_/*E*_0_ = 0.5 and *E*_∞_/*E*_0_ = 0.25, respectively. Increasing levels of grey colour of the markers for the numerical data correspond to increasing values of $\epsilon_{\mathrm{de}}$; however, the colour code and line styles in (*b*) for the analytical results are consistent with figure 3*b*.

As shown in figure 4, the presence of a process zone in CZM might influence the transition of different delamination mechanisms. We can compare the predictions from our analytical methods, which use only fracture toughness, with the FEM simulations only in the regime where the FEM data collapse to a flat line corresponding to a constant parameter for *a*. In this regime, fracture is dominated by energy, and the cohesive strength has negligible effect. Outside this regime, the strength might play a significant role and hence the scaling law $\epsilon_{c}=a\lambda^{2}G/B_{p}$ cannot be used to interpret the FEM data. As the scope of this paper is on the wrinkle-induced delamination mechanism of a viscoleastic foulant layer, focus on determination of the transition between these delamination mechanisms in FEM simulations will be presented in a separate publication. A summary is presented in electronic supplementary material, appendix 3 to emphasize that the CZM analysis is conducted with careful attention to important numerical aspects including mesh refinement, process zone length, strength and energy dominated regimes [25,29,31–37]. Our simulation results for the delamination of a viscoelastic foulant layer from a wrinkled surface in the energy regime are plotted in figure 5*a*. When compared with figure 3*b*, the FE data confirm a similar dependence of the critical strain on the three dimensionless control parameters. A consistent right-shift of the transition from the instantaneous response to the other regimes as $\epsilon_{\mathrm{de}}$ increases is also observed.

### 2.4. General solution

Our analytical method and simulations indicate that the delamination of a viscoelastic foulant layer from a wrinkled surface shows a complex dependence on three control parameters. We, therefore, investigate the design parameter space by deriving a general fit to collapse the simulation data for a wide range of these parameters. Equation (2.4) can be rewritten

2.5 $$\frac{\epsilon_{c}-\epsilon_{w}}{\epsilon_{\mathrm{de}}}=\frac{1}{1-\beta P_{\pm }[(\epsilon_{c}-\epsilon_{w}))/(\dot{\epsilon }\tau_{R})]}.$$

Equation (2.5) implies the dimensionless solution: $\left(\epsilon_{c}-\epsilon_{w}\right)/\left(\epsilon_{\mathrm{de}}\right)=\Pi (\dot{\epsilon }\tau_{R}/\epsilon_{\mathrm{de}},\beta )$. Note that the parameter $\dot{\epsilon }\tau_{R}/\epsilon_{\mathrm{de}}$ is just the inverse of the parameter *τ*_de_/*τ*_*R*_ that is shown in equation (2.2) to control the instability. Similarly, equation (2.5) can be written as: $(1/\beta )(1-(\epsilon_{\mathrm{de}}/\left(\epsilon_{c}-\epsilon_{w}\right)))=$ $P_{\pm }[(\epsilon_{c}-\epsilon_{w})/(\dot{\epsilon }\tau_{R})]$. Thus, now we can replace the general dimensionless solution with

2.6 $$\frac{1}{\beta }\left(1-\frac{\epsilon_{\mathrm{de}}}{\epsilon_{c}-\epsilon_{w}}\right)=P_{\pm }\left[\Pi \left(\frac{\dot{\epsilon }\tau_{R}}{\epsilon_{\mathrm{de}}},\beta \right)\right],$$

which predicts the collapse of all the data into a region defined by the bounded functions 0 < *P*_±_ < 1. With the use of this general solution, all data for the analytical method can be collapsed to two general curves corresponding to either the use of *W* or *U*, as shown in figure 5*b*. FEM data are more noisy due to various numerical factors in the simulations such as the determination of the onset of delamination; nevertheless, they also nicely collapse into the small area bounded by the two analytical solutions. The FEM data also show that the amount of energy available to drive fracture in the simulations is bounded by the two limiting cases considered in the analytical approaches. The upper-bound and lower-bound curves are physically intuitive as the stored energy provides the least amount of possible energy while the total work provides the largest possible energy available to drive fracture. As discussed above, we conducted an FEM analysis using an explicit solver. We controlled the dynamic effect to be small to represent a quasi-static condition; however, certain amount of dynamic energy due to the solution process and dynamic propagation of the crack tip may still be present in these highly nonlinear simulations, contributing to a difference in the amount of available energy in the simulations as compared with either limits in the analytical approach. Though neither of them exactly overlaps the numerical data from analytical methods, they provide good insight into the controlling factors in this system, and how to use them to control the delamination process. The general collapse obtained in figure 5*b* further confirms a reasonable agreement between the theoretical and numerical predictions in how the wrinkle-induced delamination mechanism of the viscoelastic foulant layer is controlled by different physical parameters related to the viscoelastic properties and loading process. Equation (2.6) shows that the three physical parameters can further be combined into two dimensionless parameters, $\dot{\epsilon }\tau_{R}/\epsilon_{\mathrm{de}}$ and *β*, significantly reducing the design parameter space needed for optimizing this complex system.

## 3. Discussion

Topography-driven surface renewal is a powerful new mechanism to drive interfacial fracture over large surfaces at risk of fouling. The initial theory was limited to an elastic response regime for the fouling layer [8]. Nevertheless, the mechanism shows promising applications in medical device design, specifically anti-thrombotic vascular grafts [9,10]. The application of actuation into medical devices requires precise knowledge of target strains, which will inform the choice of graft materials, sources of actuation loading, and range of biofoulants that the mechanism will effectively remove from the surface. In this paper, we substantially enrich the existing theory on actuating topography to account for both dynamics in the surface (strain rate $\dot{\epsilon }$) and the viscoelastic nature of the fouling layer. The results of this paper are directly applicable to the design of actuating biomedical devices targeting viscoelastic foulants such as thrombus, bacteria and biofilms [6,7,9,10].

In summary, our FE results show consistent agreements with analytical predictions of the nonlinear interplay between the three control parameters, which suggest several conditions to promote delamination in the viscoelastic regime. With the same rate of loading and adhesion energy, the critical strain for delamination onset decreases with increasing relaxation time of the foulant layer *τ*_*R*_. In other words, a foulant layer that relaxes slowly is easier to de-adhere. The limit of very fast relaxation, in which the thin foulant layer approaches the fluid-like limit, needs further investigation, as FE simulations in this regime indicate that another mechanism involving the critical strength, rather than only fracture toughness, may play a role here. With the same adhesion energy and same relaxation time, increasing loading rate $\dot{\epsilon }$ reduces the critical strain for the wrinkle-induced delamination onset. Furthermore, with the same relaxation time and rate of loading, decreasing adhesion energy also decreases the critical strain for the wrinkle-induced delamination onset. In addition, comparisons are also made for the energy balance approach using the stored energy and the total energy, and a general collapse of simulations data to a bounded regime between these analytical predictions helps to reduce the design parameter space. Thus, this work provides a first attempt towards understanding the delamination of realistic viscoelastic biofoulants.

Ultimately, topographic surface renewal is based on an energy release mechanism whereby external loading and deformation of an adherent foulant leads to accumulated energy in the foulant, which beyond a critical value is able to drive interfacial crack propagation. In the purely elastic case, as shown in the key result of our prior work (see equation (2) in Pocivavsek *et al.* [8]), the critical strain density needed to drive surface renewal is given by $\epsilon_{c}/\lambda^{2}∼1/\ell_{\mathrm{ec}}^{2}$, where $\epsilon_{c}$ is the critical nominal compressive strain in the bilayer substrate driving surface wrinkling and foulant deformation and ℓ_ec_ = (*B*_0_/*G*)^1/2^ is the elasto-capillary length scale. Because energy dissipation only occurs with interfacial fracture, the elastic case is independent of loading history; as such it is independent of actuation in the dynamic sense.

Unlike in the elastic case, the presence of viscoelasticity in the foulant introduces a second intrinsic mode of dissipation in addition to interfacial fracture. Furthermore, the strain state of a viscoelastic foulant layer is a superposition of states because of the intrinsic material softening triggered by loading. The coupling of the nonlinear, wrinkle-induced strain field with the nonlinear dependence of the strain state on loading leads to a highly nonlinear equation to determine the critical delamination strain for a viscoelastic foulant layer. We show that this critical strain is controlled by three physical quantities: the magnitude of foulant layer relaxation *E*_∞_/*E*_0_, the rate of material relaxation relative to loading rate $\dot{\epsilon }\tau_{R}$ and the critical strain to delaminate the foulant layer in the instantaneous elastic limit $\epsilon_{\mathrm{de}}∼\lambda^{2}G/B_{0}$. A fourth dimensionless parameter is the strain to initiate wrinkling $\epsilon_{w}$, which can be tuned by device construction and made negligibly small in the case of vascular graft design [3,4,9,10]. In the first part of this paper, we show that viscoelasticity, characterized as the decay of foulant stiffness from *E*_0_ to *E*_∞_ over a characteristic time scale *τ*_*R*_, breaks the critical strain for topographic de-adhesion into two limiting regimes: $\epsilon_{\mathrm{de}}\leq \epsilon_{c}-\epsilon_{w}\leq (E_{0}/E_{\infty })\epsilon_{\mathrm{de}}$. The smallest strain is set by the elastic limit [8]. However, the critical strain increases proportionally to the degree of degradation in stiffness to an upper bound set by *E*_0_/*E*_∞_. This inequality can be directly obtained from our general solution by solving for $\epsilon_{c}$ in equation (2.6) or equivalently by using figure 5*b* to determine the value of $f=P_{\pm }[\Pi ((\dot{\epsilon }\tau_{R}/\epsilon_{\mathrm{de}}),\beta )]$

3.1 $$\epsilon_{c}=\epsilon_{\mathrm{de}}{\left[1-f\left(1-\frac{E_{\infty }}{E_{0}}\right)\right]}^{-1}+\epsilon_{w},$$

where *f* ∈ [0, 1] is the range of the vertical axis in figure 5*b*. By moving *f*, the critical strain moves between the short- and long-time limits which are connected by a highly nonlinear transition region. Equation (3.1), and equivalently figure 5*b*, provide a unified approach to optimize strategies for actuated topographic surfaces designed to remove viscoelastic foulants. The horizontal axis in figure 5*b* is controlled by $\dot{\epsilon }\tau_{R}/\epsilon_{\mathrm{de}}=(\left(\epsilon /\lambda^{2}\right)/\tau )\cdot (\tau_{R}\ell_{\mathrm{ec}}^{2})$, where *τ* is the applied actuation time. The first set of parentheses contains parameters under direct control of the device designer, and the second set of parentheses incorporates intrinsic properties of the foulant.

An effective anti-fouling design must enforce the system to work under short time conditions to lower the critical strain to the value $\epsilon_{\mathrm{de}}$, which is achieved when *f* = 0. Physically, this is equivalent to a state where intrinsic viscoelastic dissipation has not had time to take effect and all accumulated strain energy is available to drive interfacial fracture. Following the results in figure 5*b* and equation (2.6), a sufficient condition to be in this regime is to satisfy $\dot{\epsilon }\tau_{R}/\epsilon_{\mathrm{de}}≳10$. Thus, a system designed to be strained to a value ϵ along a time scale *τ* must satisfy the condition $\epsilon /\tau ≳10\epsilon_{\mathrm{de}}/\tau_{R}$. Equivalently, the system must be designed to have a strain rate larger than $\epsilon_{\mathrm{de}}/\tau_{R}$. In terms of the elasto-capillary length scale whose value depends on the specific response of the foulant, we obtain the following optimal design condition using figure 5*b*:

3.2 $$\frac{\epsilon /\lambda^{2}}{\tau }≳10\frac{1/l_{\mathrm{ec}}^{2}}{\tau_{R}}.$$

Equation (3.2) provides the condition for optimal surface renewal for a given viscoelastic foulant.

In general, if one moves *f* up from 0, then the critical strain given by equation (3.1) increases. This is important from a design perspective as it provides a design criterion to improve the de-adhesion capability of the system if the optimal surface renewal condition cannot be achieved. For instance, if the system is forced to operate at $\dot{\epsilon }\tau_{R}/\epsilon_{\mathrm{de}}≳5$ then the largest value of *f* becomes 0.3 and the critical strain needed will be given by equation (3.1).

In topography-driven surface renewal for viscoelastic foulants, the fouling layer accumulates strain density ($\epsilon /\lambda^{2}$) over an applied actuation time (*τ*). We will take the example of vascular grafts as an illustration, although similar arguments can be constructed for any specific application. The designer has full control over all three parameters independently: ϵ is the nominal strain in the graft substrate and will be set by *E*_*s*_ and the load available in the system (for example, pulse pressure in vascular grafts [9,10]), surface wavelength *λ* is tunable via graft bilayer construction [3,4] and lastly *τ* is either set by the system (heart rate in the case of vascular grafts [9,10]) or, if externally driven, by an actuating power source (such as in soft robotic on-demand fouling-release urinary catheters [6,7]). These three parameters must combine such that the time-integrated accumulated strain density is within the limits set by purely intrinsic properties of the foulant layer: *G*, *E*_0_, *E*_∞_, *h* and *τ*_*R*_.

Actuated topographic vascular grafts (topografts) in contact with whole blood were shown to have optimal surface renewal when actuated at 1 Hz with a wavelength of 80 μm [9,10]. At a wavelength of 250 μm, the surface renewal capability was shown to be reduced by 50%. When the wavelength was increased to 1000 μm, the topography had no added effect on surface renewal (see fig. 5 in [9]). Surface renewal using just the elastic limit only predicts whether topographic delamination occurs or not, based on if the applied strain is larger than the critical threshold $\epsilon_{\mathrm{de}}$. Thus, it can be used to interpret the observations for the two wavelengths 80 and 1000 μm. However, it is not sufficient for explaining the cross-over delamination capability observed at the intermediate wavelength 250 μm. Figure 5*b* and equation (3.2) can be used to correlate all these observations. The left-hand side of equation (3.2) contains factors under control of the designer. In the case of topografts: ϵ∼0.15, *λ* = 80, 250, 1000 μm, *τ* = 1 s, so that the surface wavelength was studied as a design control parameter [9,10]. The right-hand side of equation (3.2) contains parameters intrinsic to the foulant. The whole blood thrombus is assumed to be incompressible with *E*_0_ = 10 kPa, *ν* = 0.5, *h* = 100 μm [9,48]. The fracture energy *G* can be calculated from published cohesive strength of whole blood *σ*_*c*_ ∼ 2 kPa [49]. Using the analysis outlined in §2.3 with *L*_pz_ ≈ *h*, $G=h\sigma_{c}^{2}/E_{0}=0.04\;N\;m^{-1}$ or 40 mJ m^−2^, equivalent to a rubber/water interface [50]. Whole blood thrombus viscoelasticity has been studied and noted to fit well to a second-order Prony expansion with $\tau_{R}^{1}∼1$ s and $\tau_{R}^{2}∼30$ s with weak dependence on strain rates [48]. The $\epsilon_{\mathrm{de}}=a\lambda^{2}G/B_{0}$ corresponding to the three wavelengths *λ* = 80, 250, 1000 μm are 0.0058, 0.056 and 0.9, where *B*_0_ = *E*_0_*h*^3^/12(1 − *ν*^2^) is the bending stiffness and the numerical pre-factor *a* is determined in figure 4 and electronic supplementary material, appendix 3. Combining these terms provide the values for the left-hand side $\left(\epsilon /\lambda^{2}\right)/\tau$ of equation (3.2) to be in the range of 2.3 × 10^7^ (m^2^ s)^−1^ for *λ* = 80 μm, 2.4 × 10^6^ (m^2^ s)^−1^ for *λ* = 250 μm and 1.5 × 10^5^ (m^2^ s)^−1^ for *λ* = 1000 μm. The ability of the actuating topography to function as an optimal surface release mechanism according to equation (3.2) can only occur if $\left(\epsilon /\lambda^{2}\right)/\tau$ is larger than 10*aG*/*τ*_*R*_*B*_0_. A range of the value for this right-hand side for the whole blood thrombus is from 9 × 10^6^ (m^2^ s)^−1^ for $\tau_{R}^{1}∼1$ s to 3 × 10^5^ (m^2^ s)^−1^ for $\tau_{R}^{2}∼30\;s$. In addition, with $\epsilon_{w}=0$ and *E*_∞_ = 0 kPa, the value in the vertical axis of figure 5*b* will be 0.96, 0.62 and −5 correspondingly for the three wavelengths *λ* = 80, 250, 1000 μm. With these computed properties, we can interpret the trend of the above experimental data for thrombus delamination.

For the wavelength *λ* = 1000 μm, the applied strain $\epsilon =0.15<\epsilon_{\mathrm{de}}=0.9$ is not enough to induce delamination. This is also consistent with the negative value −5 of the ratio on the vertical axis of the master curve in figure 5*b*. The applied strain ϵ=0.15 is big enough to induce delamination for the wavelength *λ* = 80 μm. Furthermore, at this smallest wavelength, the value of $\left(\epsilon /\lambda^{2}\right)/\tau =2.3\times 10^{7}\;{\left(m^{2}\;s\right)}^{-1}$ is larger than the upper bound 9 × 10^6^ (m^2^ s)^−1^ of the right-hand side for the two relaxation times, suggesting that optimal condition for delamination (equation 3.2) is always satisfied. This confirms the capability of these foulants to fail in the elastic limit when the smallest wavelength is used. The high value 0.96 of the ratio plotted in figure 5*b* further shows that the critical strain is far above the threshold to induce delamination. The interesting phenomenon at the intermediate wavelength *λ* = 250 μm is also explained with the incorporation of viscoelastic foulant behaviour. The applied strain ϵ=0.15 is still larger than the elastic delamination threshold $\epsilon_{\mathrm{de}}=0.056$, corresponding to the value 0.62 on the vertical axis of figure 5*b*. Thus, surface renewal using just the elastic limit predicts that the delamination occurs as in the optimal wavelength *λ* = 80 μm. This is not consistent with the experimental observation where a significant reduction in delamination capability is observed. However, by considering the inequality (3.2), at this wavelength *λ* = 250 μm, the left-hand side 2.4 × 10^6^ (m^2^ s)^−1^ lies in the middle of the right-hand side range 9 × 10^6^ (m^2^ s)^−1^ for $\tau_{R}^{1}∼1$ s to 3 × 10^5^ (m^2^ s)^−1^ for $\tau_{R}^{2}∼30\;s$. This suggests that whether optimal delamination is satisfied or not depends on the relaxation properties of the foulants. In addition, the intermediate value 0.62 of the ratio plotted on the vertical axis of figure 5*b* suggests that delamination may or may not occur depending on how the dissipation energy plays a role in the delamination process. Specifically, at this wavelength, the value $\dot{\epsilon }\tau_{R}/\epsilon_{\mathrm{de}}$ on the horizontal axis of figure 5*b* is 2.67 and 80 for the two relaxation times $\tau_{R}^{1}$ and $\tau_{R}^{2}$, respectively. Thus, the corresponding point on the master curve for $\tau_{R}^{1}$ lies above the critical point predicted using the total energy approach, but is below the critical point predicted using the stored energy approach. This indicates that viscous dissipation is likely to play a role in dampening the effect of topographic surface renewal at this wavelength, which can be why in practice the capability for delamination to happen is reduced at *λ* = 250 μm when compared with at the optimal wavelength *λ* = 80 μm. This analysis shows that going to even smaller wavelengths would most improve surface renewal given the nonlinear dependence on *λ*. Thus, our analysis allows a concrete, quickly applicable methodology to pick design parameters for given anti-fouling applications of topography-driven surface renewal. It shows that to optimize topographic de-adhesion for viscoelastic foulants the condition set forth in equation (3.2) should be satisfied. This helps guide design of anti-fouling surfaces targeted for biomedical applications such as anti-thrombotic grafts.

The energy balance and numerical approaches in this work and the previous publication in the elastic limit [8] provide a general framework to study the interaction between the external loading, the foulant deformation and the surface energy. Thus, it will be the basis for future studies that focus on incorporating more complexity in loading conditions, material behaviours and interfacial properties. Specifically, the relaxation behaviour of the foulant will depend on the frequency of the external loading cycle, such as the constant loading and unloading (equivalently contraction and expansion) of the arterial wall [17–19]. Hence, the energy balance must consider how the energy in the foulant is accumulated and relaxed during multiple excitations to determine the critical number of cycles to induce foulant delamination. In our model, we assume that when delamination occurs, it is an irreversible process and the detached foulant does not reattach to the arterial wall when the wall relaxes. As such, the new configuration with this new crack length has to be used in the next contracting and expanding cycle of the arterial wall. This assumption should be experimentally tested and our model can be further enriched if partial reattachment phenomena occurs. Another interesting phenomenon that can be included to extend our studies is the relaxation behaviour in the arterial wall during a cycle. This will lead to changes in the wrinkle topography where the wrinkle wavelength and amplitude are influenced by the viscoelastic properties of the wall [51,52], consequently affecting the scaling for the critical strain. Furthermore, adhesion in biological materials can be a rate-dependent process where the fracture toughness *G* depends on the loading rates [53]. This complexity, if being added to our energy balance model, will lead to the dependence of $\epsilon_{\mathrm{de}}$ on the loading rate even in the elastic foulant limit. When the foulant is viscoelastic, we anticipate another dimensionless parameter relating how fast the foulant relaxes to how fast *G* changes with loading will also become a control parameter. For the numerical approach, a rate-dependent cohesive zone model with mixed-mode failure conditions can be developed and incorporated into our model to tackle more complex interfacial delamination processes. Additionally, the flat configuration in this work is applicable to study the limit when the length of the foulant, which covers multiple wrinkle wavelengths, is much smaller than the radius of the arterial wall. The curvature of the artery can be conveniently introduced into our model as a next step to study its effect on the delamination strain when this limit does not apply.

## Data Availability

Data for this study can be found in the main manuscript and the electronic supplementary material files and video [54].

## References

[1] Li D, Zheng Q, Wang Y, Chen H. 2014 Combining surface topography with polymer chemistry: exploring new interfacial biological phenomena. Polym. Chem. **5**, 14-24. (10.1039/C3PY00739A)

[2] Bixler GD, Bhushan B. 2012 Biofouling: lessons from nature. Phil. Trans. R. Soc. A **370**, 2381-2417. (10.1098/rsta.2011.0502) 22509063

[3] Genzer J, Groenewold J. 2006 Soft matter with hard skin: from skin wrinkles to templating and material characterization. Soft Matter **2**, 310-323. (10.1039/b516741h) 32646128

[4] Pocivavsek L, Leahy B, Holten-Andersen N, Lin B, Lee KYC, Cerda E. 2009 Geometric tools for complex interfaces: from lung surfactant to the mussel byssus. Soft Matter **5**, 1963-1968. (10.1039/b817513f)

[5] Russell TP. 2002 Surface responsive materials. Science **297**, 964-967. (10.1126/science.1075997) 12169722

[6] Shivapooja P, Wang Q, Orihuela B, Rittschof D, López GP, Zhao X. 2013 Bioinspired surfaces with dynamic topography for active control of biofouling. Adv. Mater. **25**, 1430-1434. (10.1002/adma.v25.10) 23292960

[7] Levering V, Wang Q, Shivapooja P, Zhao X, López GP. 2014 Soft robotic concepts in catheter design: an on-demand fouling-release urinary catheter. Adv. Healthc. Mater. **3**, 1588-1596. (10.1002/adhm.v3.10) 24668920 PMC4176551

[8] Pocivavsek L, Pugar J, O’Dea R, Ye S, Wagner W, Tzeng E, Velankar S, Cerda E. 2018 Topography-driven surface renewal. Nat. Phys. **3**, 948-953. (10.1038/s41567-018-0193-x) PMC11271749 39055780

[9] Pocivavsek L, Ye S, Pugar J, Tzeng E, Cerda E, Velankar S, Wagner WR. 2019 Active wrinkles to drive self-cleaning: a strategy for anti-thrombotic surfaces for vascular grafts. Biomaterials **192**, 226-234. (10.1016/j.biomaterials.2018.11.005) 30458358 PMC7248685

[10] Nath NN, Pocivavsek L, Pugar JA, Gao Y, Salem K, Pitre N, McEnaney R, Velankar S, Tzeng E. 2020 Dynamic luminal topography: a potential strategy to prevent vascular graft thrombosis. Front. Bioeng. Biotechnol. **8**, 573400. (10.3389/fbioe.2020.573400) 32984298 PMC7487362

[11] Svendsen E, Tindall AR. 1988 The internal elastic membrane and intimal folds in arteries: important but neglected structures? Acta. Physiol. Scand. Suppl. **572**, 1-71. 3232528

[12] Nguyen N, Nath N, Deseri L, Tzeng E, Velankar SS, Pocivavsek L. 2020 Wrinkling instabilities for biologically relevant fiber-reinforced composite materials with a case study of neo-Hookean/Ogden–Gasser–Holzapfel bilayer. Biomech. Model. Mechanobiol. **19**, 2375-2395. (10.1007/s10237-020-01345-0) 32535739 PMC7920575

[13] Hasan J, Chatterjee K. 2015 Recent advances in engineering topography mediated antibacterial surfaces. Nanoscale **7**, 15 568-15 575. (10.1039/C5NR04156B) 26372264 PMC4642214

[14] Chen L, Han D, Jiang L. 2011 On improving blood compatibility: from bio-inspired to synthetic design and fabrication of biointerfacial topography at micro/nano scales. Colloids Surf. B **85**, 2-7. (10.1016/j.colsurfb.2010.10.034) 21106352

[15] Mao C, Liang C, Luo W, Bao J, Shen J, Hou X, Zhao W. 2009 Preparation of lotus-leaf-like polystyrene micro- and nanostructure films and its blood compatibility. J. Mater. Chem. **19**, 9025-9029. (10.1039/b912314h)

[16] Koh LB, Rodriguez I, Venkatraman SS. 2010 The effect of topography of polymer surfaces on platelet adhesion. Biomaterials **31**, 1533-1545. (10.1016/j.biomaterials.2009.11.022) 19945746

[17] Shaw T, Winston M, Rupp CJ, Klapper I, Stoodley P. 2004 Commonality of elastic relaxation times in biofilms. Phys. Rev. Lett. **93**, 098102. (10.1103/PhysRevLett.93.098102) 15447143

[18] Christensen RM. 2003 Theory of viscoelasticity. New York, NY: Dover Publications.

[19] Wineman AS, Rajagopal KR. 2000 Mechanical response of polymers. Cambridge, UK: Cambridge University Press.

[20] Kinloch AJ, Lau CC, Williams JG. 1994 The peeling of flexible laminates. Int. J. Fract. **66**, 45-70. (10.1007/BF00012635)

[21] Hutchinson JW, Suo Z. 1992 Mixed mode cracking in layered materials. Adv. Appl. Mech. **29**, 63-191. (10.1016/S0065-2156(08)70164-9)

[22] Begley MR, Hutchinson JW. 2017 The mechanics and reliability of films, multilayers, and coatings (chs 4 and 9). Cambridge, UK: Cambridge University Press.

[23] Vella D, Bico J, Boudaoud A, Roman B, Reis PM. 2009 The macroscopic delamination of thin films from elastic substrates. Proc. Natl Acad. Sci. USA **106**, 10 901-10 906. (10.1073/pnas.0902160106) 19556551 PMC2708712

[24] Oshri O, Balazs AC. 2018 Delamination of a thin sheet from a soft adhesive Winkler substrate. Phys. Rev. E **97**, 062803. (10.1103/PhysRevE.97.062803) 30011476

[25] Davidson P, Waas AM. 2012 Non-smooth mode I fracture of fibre-reinforced composites: an experimental, numerical and analytical study. Phil. Trans. R. Soc. A **370**, 1942-1965. (10.1098/rsta.2011.0381) 22431765

[26] Chai H, Babcock CD, Knauss WG. 1981 One dimensional modelling of failure in laminated plates by delamination buckling. Int. J. Solids Struct. **17**, 1069-1083. (10.1016/0020-7683(81)90014-7)

[27] Chen H, Feng X, Huang Y, Huang Y, Rogers JA. 2013 Experiments and viscoelastic analysis of peel test with patterned strips for application to transfer printing. J. Mech. Phys. Solids **61**, 1737-1752. (10.1016/j.jmps.2013.04.001)

[28] Srinivas MV, Ravichandran G. 1994 Interfacial crack propagation in a thin viscoelastic film bonded to an elastic substrate. Int. J. Fract. **65**, 31-47. (10.1007/BF00017141)

[29] Golovin K, Dhyani A, Thouless MD, Tuteja A. 2019 Low–interfacial toughness materials for effective large-scale deicing. Science **364**, 371-375. (10.1126/science.aav1266) 31023920

[30] Mei H, Landis CM, Huang R. 2011 Concomitant wrinkling and buckle-delamination of elastic thin films on compliant substrates. Mech. Mater. **43**, 627-642. (10.1016/j.mechmat.2011.08.003)

[31] Turon A, Davila CG, Camanho PP, Costa J. 2007 An engineering solution for mesh size effects in the simulation of delamination using cohesive zone models. Eng. Fract. Mech. **74**, 1665-1682. (10.1016/j.engfracmech.2006.08.025)

[32] Parmigiani JP, Thouless MD. 2007 The effects of cohesive strength and toughness on mixed-mode delamination of beam-like geometries. Eng. Fract. Mech. **74**, 2675-2699. (10.1016/j.engfracmech.2007.02.005)

[33] Heinrich C, Waas AM. 2012 Investigation of progressive damage and fracture in laminated composites using the smeared crack approach. In *53rd AIAA/ASME/ASCE/AHS/ASC Structures, Structural Dynamics and Materials Conf., Honolulu, HI*. Reston, VA: American Institute of Aeronautics and Astronautics.

[34] Xie D, Waas AM. 2006 Discrete cohesive zone model for mixed-mode fracture using finite element analysis. Eng. Fract. Mech. **73**, 1783-1796. (10.1016/j.engfracmech.2006.03.006)

[35] Nguyen N, Waas AM. 2016 A novel mixed-mode cohesive formulation for crack growth analysis. Compos. Struct. **156**, 253-262. (10.1016/j.compstruct.2015.11.015)

[36] Lin S, Nguyen N, Waas AM. 2019 Application of continuum decohesive finite element to progressive failure analysis of composite materials. Compos. Struct. **212**, 365-380. (10.1016/j.compstruct.2019.01.021)

[37] Nguyen N, Waas AM. 2017 Continuum decohesive finite element modeling of fiber-reinforced polymer composites: mesh-objectivity and sensitivity studies. In *58th AIAA/ASCE/AHS/ASC Structures, Structural Dynamics and Materials Conf., Grapevine, TX*. Reston, VA: American Institute of Aeronautics and Astronautics.

[38] Allen HG. 1969 Analysis and design of structural sandwich panels. Oxford, UK: Pergamon Press.

[39] Bowden N, Brittain S, Evans AG, Hutchinson JW, Whitesides GM. 1998 Spontaneous formation of ordered structures in thin films of metals supported on an elastomeric polymer. Nature **393**, 146-149. (10.1038/30193)

[40] Pocivavsek L, Dellsy R, Kern A, Johnson S, Lin B, Lee KYC, Cerda E. 2008 Stress and fold localization in thin elastic membranes. Science **320**, 912-916. (10.1126/science.1154069) 18487188

[41] Sun J, Xia S, Moon M, Oh KH, Kim K. 2012 Folding wrinkles of a thin stiff layer on a soft substrate. Proc. R. Soc. A **468**, 932-953. (10.1098/rspa.2011.0567) PMC3637001 23633907

[42] Cao Y, Hutchinson JW. 2012 Wrinkling phenomena in neo-Hookean film/substrate bilayer. J. Appl. Mech. **79**, 031019. (10.1115/1.4005960)

[43] Cerda E, Mahadevan L. 2003 Geometry and physics of wrinkling. Phys. Rev. Lett. **90**, 074302. (10.1103/PhysRevLett.90.074302) 12633231

[44] Bazant ZP, Cedolin L. 2010 Stability of structures: elastic, inelastic, fracture and damage theories. Singapore: World Scientific Publishing.

[45] Poole RJ. 2012 The Deborah and Weissenberg numbers. Rheol. Bull. **53**, 32-39.

[46] Lawn B. 1993 Fracture of brittle solids. Cambridge, UK: Cambridge University Press.

[47] Dassault Systèmes. 2018 Abaqus user’s manual, ver. 6.18 [online]. MA, USA.

[48] Sugerman GP, Parekh SH, Rausch MK. 2020 Nonlinear, dissipative phenomena in whole blood clot mechanics. Soft Matter **16**, 9908-9916. (10.1039/D0SM01317J) 33029598

[49] Chan KYT, Zhao C, Siren EMJ, Chan JCY, Boschman J, Kastrup CJ. 2016 Adhesion of blood clots can be enhanced when copolymerized with a macromer that is crosslinked by coagulation factor XIIIa. Biomacromolecules **17**, 2248-2252. (10.1021/acs.biomac.6b00481) 27140446 PMC5496764

[50] de Gennes P-G. 1998 Simple views on condensed matter physics. Modern Condensed Matter Physics, vol. 8. London, UK: World Scientific Publishing.

[51] Guan X, Sarma AP, Hamesh EK, Yang J, Nguyen N, Cerda E, Pocivavsek L, Velankar SS. 2022 Compression-induced buckling of thin films bonded to viscous substrates: uniform wrinkles vs localized ridges. Int. J. Solids Struct. **254–255**, 111843. (10.1016/j.ijsolstr.2022.111843)

[52] Im SH, Huang R. 2005 Evolution of wrinkles in elastic-viscoelastic bilayer thin films. J. Appl. Mech. **72**, 955-961. (10.1115/1.2043191)

[53] Han G, Eriten M, Henak CR. 2020 Rate-dependent adhesion of cartilage and its relation to relaxation mechanisms. J. Mech. Behav. Biomed. Mater. **102**, 103493. (10.1016/j.jmbbm.2019.103493) 31634661

[54] Nguyen N, Hamm Hahn E, Velankar S, Cerda E, Pocivavsek L. 2023 Topographic de-adhesion in the viscoelastic limit. Figshare. (10.6084/m9.figshare.c.6350453) PMC9832294 36628528

